# Establishment of a Lentiviral Vector Encoding Human HGF and the Infection of Human ADSCs

**DOI:** 10.1155/2013/724896

**Published:** 2013-01-20

**Authors:** Xiaoyu Zhu, Lei Xu, Xin Liu, Jingsheng Wu, Weibo Zhu, Xiaoyan Cai, Zimin Sun

**Affiliations:** ^1^Department of Hematology, Anhui Provincial Hospital of Anhui Medical University, Hefei 23001, China; ^2^Department of Osteology, Anhui Provincial Hospital of Anhui Medical University, Hefei 23001, China

## Abstract

The delivery of adipose-derived stem cells (ADSCs) for promoting tissue repair has become a potential new therapy, while hepatocyte growth factor (HGF) is an important growth factor with angiogenic, anti-fibrotic, and anti-inflammatory benefits. In this paper, hADSCs were separated, cultured and identified based on the expression of cell surface antigens and multiple differentiation potential. We successfully generated a lentiviral vector encoding human HGF, infected hADSCs with this vector and examined the protein expression pattern. Finally we found that the hHGF lentiviral vector was successfully generated, and the lentiviral vector was able to safely infect hADSCs with high infection efficiency, thereby producing cells that overexpressed hHGF, which may provide a new strategy for the treatment of ischemic heart disease (IHD) and other ischemic diseases.

## 1. Introduction 

Ischemic heart disease (IHD) remains the leading cause of death in modern society. The pathologic mechanism of IHD is characterized by the irreversible loss of functional cardiomyocytes followed by myocardial fibrosis and ventricular remodeling due to the decreased number of vessels, which diminishes the blood supply [1]. Therapeutic angiogenesis is currently a popular focus of study in IHD research involving growth factor protein therapy, cell transplantation, and gene therapy [2]. With the development of vascular tissue engineering, stem cell technology has been widely used and represents the latest advances in this field. Mesenchymal stem cells (MSCs) are a group of heterogeneous multipotent cells that can be isolated from many tissue types. Many studies have focused on MSCs isolated from bone marrow. However, there are issues associated with the clinical use of MSCs derived from bone marrow, including pain, morbidity, and low numbers of harvested cells. In contrast, adipose tissue contains an abundance of adult stem cells (termed adipose-derived stem cells, ADSCs), and these ADSCs are easy to isolate and differentiate into osteogenic, chondrogenic, myogenic, endothelial, and neurogenic lineages [3, 4]. Animal studies have shown that ADSCs display potential beneficial effects for therapeutic angiogenesis [5, 6].

 HGF, which was originally identified and cloned for hepatocyte, has been reported to exert mitogenic, angiogenic, antiapoptotic, and antifibrotic activity in various cell types [7]. Several studies have revealed that HGF is an endogenous cardioprotective factor, as it protects cardiomyocytes from acute ischemic death during acute myocardial infarction (AMI) and enhances the survival of cardiomyocytes exposed to oxidant stress [8, 9]. Recent study showed that intramyocardial injection ofHGFand microbubbles (MBs) in combination with insonation enhanced neovascularization and reduced ventricular remodeling and infarct size [10].

Therefore, the angiogenic effects of ADSC transplantation combined with HGF expression directed into the infracted heart area may exert more beneficial effects than either gene therapy or stem cell therapy alone. In the present study, we generated a lentiviral vector encoding human HGF, infected ADSCs with this vector, and examined the protein expression pattern to provide a new strategy for the treatment of IHD. 

## 2. Materials and Methods

### 2.1. Identification and Sequencing of the pcDNA3.0-hHGF and hHGF PCR Amplification Products

 The pcDNA3.0 plasmid encoding human HGF (pcDNA3.0-hHGF) was a kind gift provided by Dr. Y. S. Zhou at the Chinese Academy of Military Medical Sciences. The pcDNA3.0 plasmid and hHGF open reading frame (ORF) sequences were obtained from the Invitrogen and NCBI websites. Based on the genomic sequences, we selected the Hind III, Xba I, BamH I, and Not I restriction enzyme sites, and we sequenced the DNA. The hHGF mRNA primers were designed using Primer 3 software. The forward primer (5′-GATCCGCTAGCGCTACCGGTCGCCACC*ATGTGGGTGACCAAACTCC*-3′) and reverse primer (5′-TCACCATGGTGGCGACCGGTAG*TGACTGTGGTACCT TATATGTTA*-3′) both contained an Age I restriction enzyme site. The length of the amplified segment was 2218 bp.

The PCR products were assessed via electrophoresis using a 1% agarose gel. The PCR products were isolated from the gel using a viltaLight lamp. The target gene PCR products were isolated and purified using a gel extraction kit.

### 2.2. Cloning the hHGF Genomic Fragment into the pGC-E1 Vector

 The purified hHGF fragment and pGC-E1 gene plasmid were separately digested with Age I. Using an In-Fusion Kit, the hHGF fragment was ligated into the pGC-E1 expression vector, which was previously digested with Age I. The total ligation reaction volume was 20 mL (2 mL of 100 mg/mL vector DNA, 2 mL of 100 ng/mL hHGF fragment, 1 mL of 10x In-Fusion exchange enzyme buffer, 0.5 mL of In-Fusion exchange enzyme, and 13.5 mL of ddH_2_O). Two control groups, either without vector DNA or without hHGF, were included in the reaction system. The reaction was run at 23°C for 15 min followed by 42°C for 15 min, and the DNA cloning ligation system was then prepared. 

 After transforming the DNA into DH5*α* competent bacteria cells, which were grown in LB medium, the positive clones were identified via PCR to verify the successful insertion of the hHGF fragment into the lentiviral shuttle plasmid, and the PCR products were subsequently analyzed via DNA sequencing by Beijing GeneChem Co. The final plasmid was termed pGC-E1-hHGF. 

### 2.3. Production and Titration of the Recombinant Lentiviral Vector

The recombinant pGC-E1-hHGF vector and two packaging components (pHelper1.0 and pHelper2.0) were extracted from the positively transformed bacteria. The plasmid DNA concentration was measured using UV A260/A280 absorption values (normally between 1.8 and 2.0). The recombinant lentiviral vector, Lenti-hHGF, was generated by cotransfecting 293T cells with 20 g of pGC-E1-hHGF, 15 g of pHelper1.0, and 10 g of pHelper2.0 in 10 cm dishes with Lipofectamine 2000 (Invitrogen, USA). The 293T cells were then cultured in DMEM (Gibco, Invitrogen) containing 10% heat-inactivated fetal bovine serum FBS (Gibco, Invitrogen). The culture supernatants were collected every 24 h for 3 days, filtered through a 0.45 um pore size filter, and concentrated twice via ultracentrifugation at 50,000 xg at 20°C for 120 min. The viral supernatants were concentrated 1,000 times by ultracentrifugation, resuspended in sterile phosphate-buffered saline (PBS), and then stored at −80°C until use. The virus titers were determined via a one-in-one whole dilution.

### 2.4. Isolation, Culture, and Identification of ADSCs 

#### 2.4.1. ADSC Isolation and Culture

Human adipose tissue derived from patients undergoing selective section-assisted lipectomy was collected, after obtaining informed consent from the patients, according to procedures approved by the Ethics Committee at Chinese Academy of Medical Sciences and Anhui Provincial Hospital of Anhui Medical University. This tissue collection procedure has been described previously [1]. Briefly, adipose tissue was extensively washed with D-Hanks' solution (Gibco Life Technologies, Paisley, UK) to remove contaminating debris and red blood cells, cut into small pieces, and then digested with 0.2% collagenase II (Sigma, St. Louis, MO, USA) at 37°C for 30 min with gentle agitation. The collagenase was inactivated with an equal volume of DMEM/10% FBS. The cells were washed twice and plated in T-75 tissue culture flasks at a density of 2 × 10^6^ cells/mL. The expansion medium contained 90% DMEM, 10% FBS, 10 ng/mL epidermal growth factor (EGF, Sigma), 10 ng/mL platelet-derived growth factor BB (PDGF-BB, Sigma), 100 U/mL penicillin, and 100 mg/mL streptomycin (Gibco, Invitrogen). The medium was changed 24 h later to remove the nonadherent cells. Once the adherent cells were more than 90% confluent, the cells were separated and cultured. 

#### 2.4.2. Immunophenotype Analysis of ADSCs

The third passage of ADSCs was adopted for immunophenotype analysis. Cells (2 × 10^5^) were resuspended in 200 *μ*L of PBS containing 0.5% BSA and incubated (in the dark) for 30 min at 4°C with fluorescence-labeled antibodies against human CD14-phycoerythrin (PE), CD29-fluorescein isothiocyanate (FITC), CD31-FITC, CD34-PE, CD44-FITC, CD45-peridinin chlorophyll protein (PerCP), CD71-PE, CD86-PerCP, CD106-PE, CD117-PE, HLA-DR-FITC, or the appropriate isotype controls, which were obtained from BD Biosciences Pharmingen (Franklin Lakes, NJ, USA). The cells were analyzed via flow cytometry using an ELITE flow cytometer with WinMDI2.9 software (Beckman Coulter, Fullerton, CA,USA).

#### 2.4.3. The Multiple Differentiation Ability of ADSCs

For osteogenic differentiation, cells at passage 5 were incubated in DMEM medium containing 10% FBS, 20 nM dexamethasone, 100 U/mL penicillin, 100 mg/mL streptomycin, 2.5 mg/mL amphotericin, 10 mM b-glycerophosphate, and 0.05 mM L-ascorbic acid-2- phosphate. Control cultures were fed only DMEM containing 10% FBS and antibiotics. After 21 days of culture, the osteogenic differentiation of stem cells was confirmed via the positive alizarin red staining of the mineralized matrix.

 For adipogenic differentiation, the cells were incubated with DMEM medium containing 3% FBS, antibiotics, 33 mM biotin, 17 mM pantothenic acid, 1 mM insulin, 1 mM dexamethasone, 0.5 mM 3-isobutyl-1-methylxanthine (IBMX), 5 mM rosiglitazone, and 5% rabbit serum for 3 days. The cells were then treated with inducing medium without rosiglitazone and IBMX. After 19 days of culture, the cells were fixed with 10% formalin and incubated for 20 min with Oil-Red O to visualize lipid droplets.

### 2.5. Infection of ADSCs with Lentiviral Vectors

 On the day of infection, the cells were plated at a density of 4 × 10^4^ cells/well in 96-well plates along with lenti-hHGF or lenti-GFP at different multiplicities of infection (MOI) in serum-free growth medium containing 5 *μ*g/mL polybrene. Serum-containing growth medium was added after 4 h and replaced after 48 h. Reporter gene expression was examined using fluorescent microscopy on day 4 or 5 after infection. The ideal MOI for the formal experiment was selected. The infected cells were passaged, and the percentage of GFP^+^ cells was assessed via flow cytometry.

### 2.6. Detection of hHGF Protein in Target Cells

The hHGF protein expression levels in the ADSCs were assessed via western blot analysis. Briefly, uninfected ADSCs and ADSCs infected with either lenti-hHGF or lenti-GFP were harvested, and a specific volume of ice cold 2x lysis buffer was applied to the cells. The supernatant was collected, and the protein concentration was assessed using a Bradford assay. Sample buffer (2x) was then added to the samples (the volume of buffer depended on the concentration of the protein sample), and the proteins were denatured at 100°C for 5 minutes. The protein samples were then separated via 12% SDS-PAGE electrophoresis (25 *μ*g/pore). The proteins were transferred to PVDF membranes at 120 V for approximately 2 hours. Subsequently, the membrane was blocked with a 5% milk TBST solution overnight. The membranes were then incubated with primary antibody (1 : 1000 dilution) at room temperature for 2 hours, washed three times with TBST (3 × 10 min), incubated with secondary antibody (1 : 1000 dilution) at room temperature for 2 hours, and washed again. Finally, ECL reagent was added to visualize the protein bands. The film was exposed after a 5-minute incubation with ECL reagent, and the film was then developed and fixed. Actin was used as a control to assess the relative expression levels of the proteins in each group. 

To detect the expression of hHGF protein in supernatant, ELISA was used. Cell culture supernatants were collected from the culture media of uninfected ADSCs and ADSCs infected with either lenti-hHGF or lenti-GFP at the time points of 1, 3, 5 and 7 days after infection seperately. Concentrations of hHGF protein in the cell supernatant were determined by ELISA (Human HGF ELISA kit; R&D) according to the manufacturer's protocols.

### 2.7. Statistical Analysis

SPSS 16.0 for windows was used for all statistical analysis. Variables were presented as mean ± standard deviation. Comparisons between 2 groups were made by Student's *t*-test. For ≥3 groups, one-way analysis of variance with a post hoc test of LSD test was used for the statistical analysis. A *P* value of <0.05 was considered significant.

## 3. Results

### 3.1. Identification and Sequencing of the pcDNA3.0-hHGF and hHGF PCR Amplification Products

**Figure 1:**
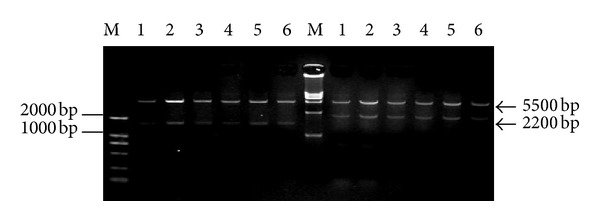
The pcDNA3.0-hHGF vector double digested, first with Hind III, Xba I and BamH I (left) and then with Not I (right). 1–6: Clone 1–6; M: DNA marker. Clones 1–6 all tested positive.

**Figure 2:**
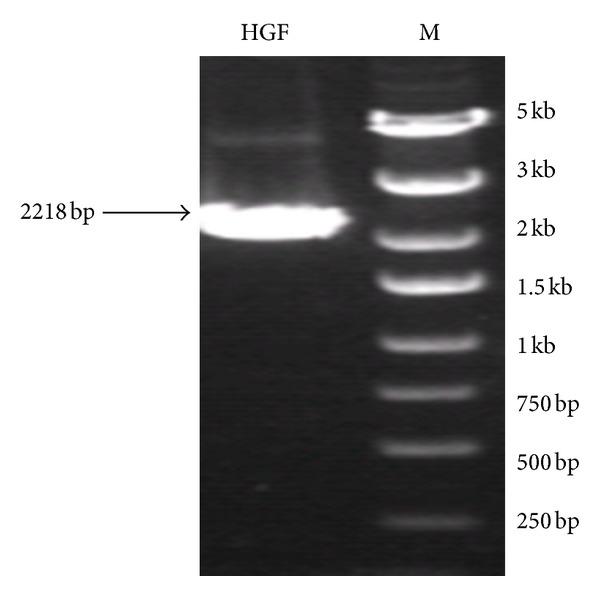
hHGF amplification product (PCR).

Six pcDNA3.0-hHGF clones were double digested, first with Hind III, Xba I, and BamH I and then with Not I. Based on the sequences after digestion with Hind III and Xba I, four bands (5.4 kb, 1.7 kb, 440 bp, and 113 bp) were identified. After digestion with BamH I and Not I, a vector band (approximately 5.5 kb) and an hHGF band (approximately 2.2 kb) were observed. According to the results, there were six positive clones with two bands (approximately 5.4 kb and 1.7 kb) after digestion with Hind III and Xba I and two bands (approximately 5.5 kb and 2.2 kb) after digestion with BamH I and Not I (Figure 1). The third clone was submitted for sequencing analysis. The base sequence of the inserted gene in the pcDNA3.0 plasmid matched the genomic sequence of hHGF provided by GenBank. As predicted, the hHGF product generated by the two primers ran at approximately 2218 bp on a gel (Figure 2). 

### 3.2. pGC-E1-hHGF Plasmid Construction and Sequencing

**Figure 3:**
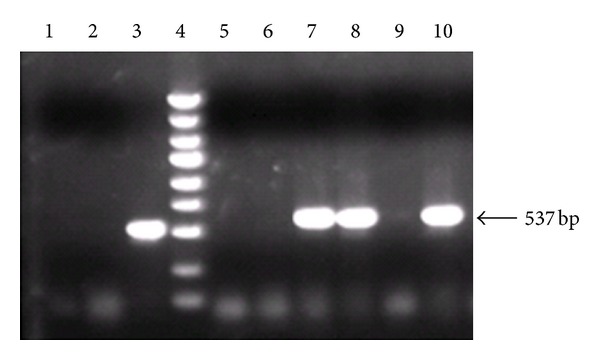
Verification of pGC-E1-hHGF expression in bacterial clones via PCR. Lane 1: negative control (ddH_2_O); lane 2: negative control (pGC-E1 empty vector); lane 3: positive control (pcDNA3.0-hHGF); lane 4: marker (5 kb, 3 kb, 2 kb, 1.5 kb, 1 kb, 750 bp, 500 bp, and 250 bp); lanes 5–10: six pGC-E1-hHGF-transformed clones. Clones 7, 8, and 10 tested positive.

**Figure 4:**
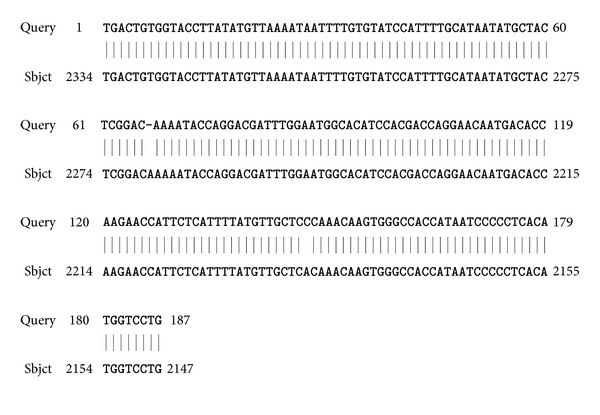
Gene sequence results of the hHGF insert in the pGC-E1-hHGF expression vector (after the positive clones were selected).

The purified hHGF fragment and the pGC-E1 gene plasmid were digested separately with Age I, and the pGC-E1-hHGF vector was generated via In-Fusion enzyme ligation. DH5*α* competent bacteria cells were transformed, and three positive bacterial clones containing the hHGF coding sequence were identified via PCR. The product size was 537 bp (Figure 3). The recombinant pGC-E1-hHGF plasmid was confirmed via sequencing, and the sequence was consistent with the hHGF gene sequence provided by GenBank (Figure 4).

### 3.3. Production and Titration of the Recombinant Lentiviral Vector

**Figure 5:**
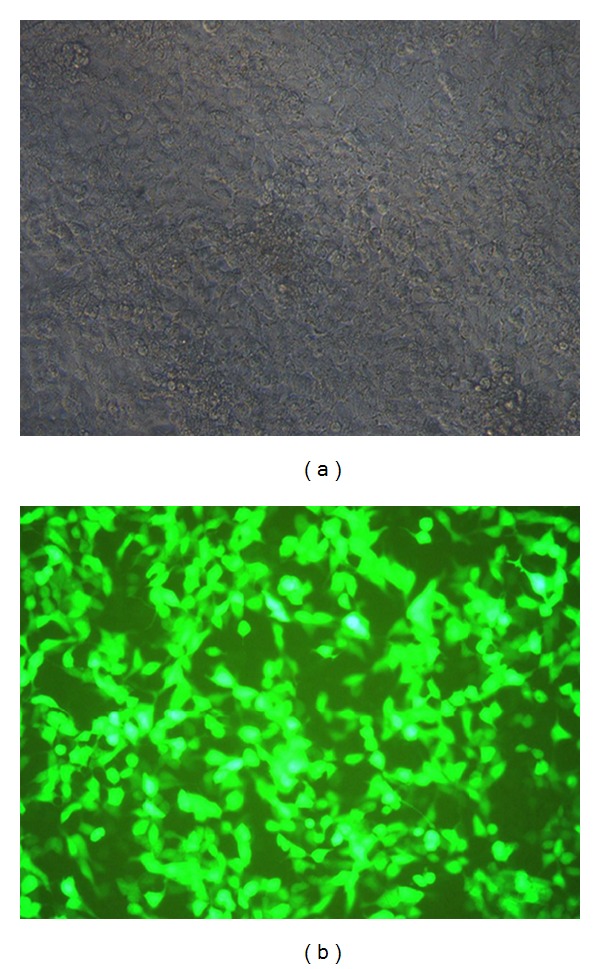
GFP expression of the hHGF recombinant lentiviral plasmid in 293T cells 48 h after transfection (100x). Left: dark field; right: bright field.

After cotransfection of 293T cells with the three plasmids, GFP expression was assessed using a fluorescence microscope to ensure that the lentiviral vectors were properly generated (Figure 5). The virus titers were determined via a one-in-one whole dilution, and the final virus titers were 1 × 10^8^ TU/mL after concentration.

### 3.4. Immunophenotype Analysis of ADSCs

**Figure 6:**
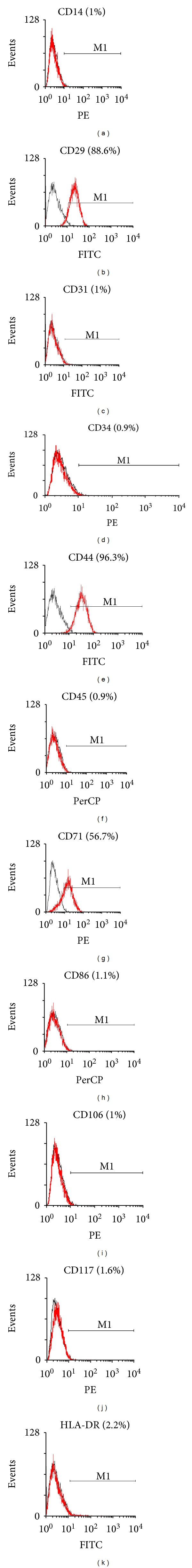
Human ADSC phenotypes at the third passage, as assessed via flow cytometry analysis. The red lines represent the specific fluorescence-labeled antibodies, and the black lines represent the isotype controls.

The freshly isolated ADSCs displayed adherence and expansion in culture, and they assumed a fibroblast-like morphology when observed using a light microscope. After the third passage, a FACS analysis revealed that the ADSCs were positive for CD29, CD44, and CD71, but the ADSCs assayed negative for hematopoietic and endothelial lineage markers, including CD31, CD34, CD45, CD106, and CD117, resulting in a phenotype similar to that of bone marrow MSCs. Moreover, immunogenicity markers, including CD14, CD45, CD86, and HLA-DR, were not observed (Figure 6).

### 3.5. Osteogenic and Adipogenic Differentiation of ADSCs

**Figure 7:**
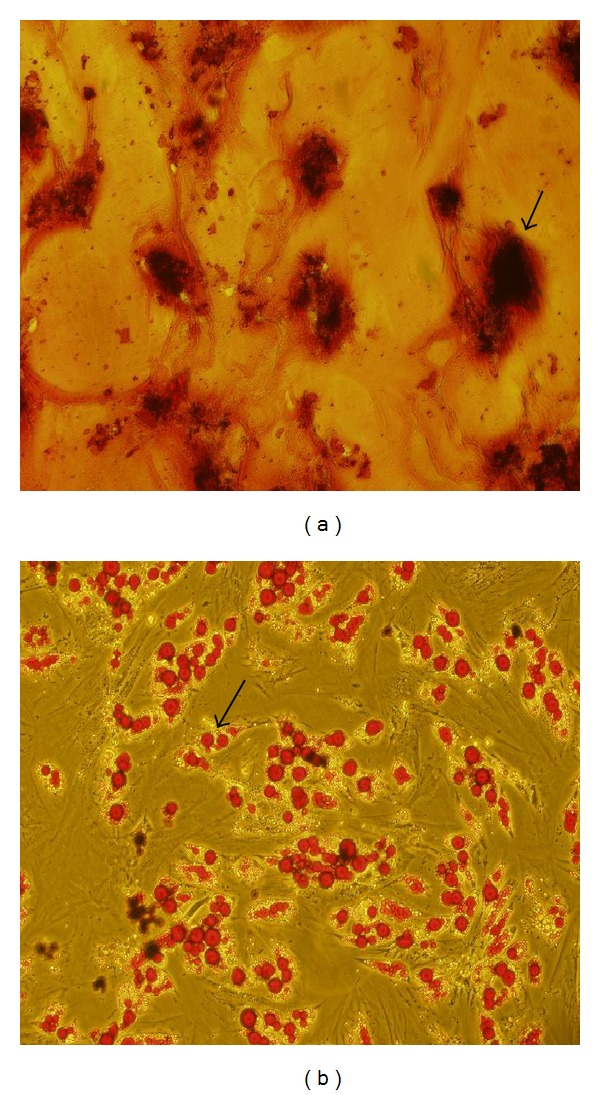
Osteogenic and adipogenic differentiation of human ADSCs. (a) ADSCs treated with osteogenic media for 21 d (stained with alizarin red). The arrow indicates the mineralized matrix produced by osteoblasts (100x). (b) ADSCs treated with adipogenic media for 21 d (stained with Oil Red-O). The arrow indicates oil droplets (100x).

The osteogenic differentiation of human ADSCs at passage 5 was confirmed via alizarin red staining (Figure 7(a)). After feeding the ADSCs with osteogenic-inducing media, dark red mineralized bone matrix (bone nodules) was observed within the alizarin red-stained section.

The adipogenic differentiation of human ADSCs was confirmed via Oil Red-O staining. After feeding the ADSCs with adipogenic-inducing media for 21 d, oil droplets were observed in the cytoplasm (Figure 7(b)).

### 3.6. The Infection Efficiency of ADSCs with Lentiviral Vectors

**Figure 8:**
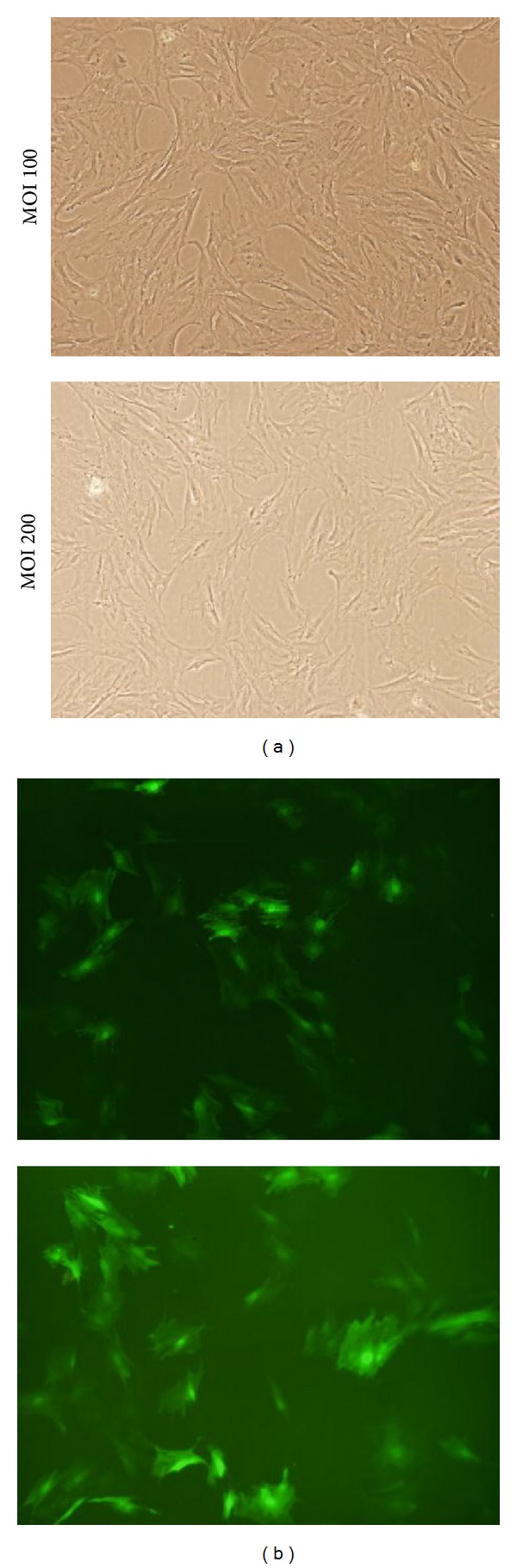
Infection of ADSCs with lenti-hHGF at an MOI of 100 or 200 (100x). GFP expression was observed via either light (a) or fluorescence microscopy (b).

On the day of infection, hADSCs were infected with either lenti-hHGF or lenti-GFP at an MOI of 100, or 200. GFP expression was observed via fluorescent microscopy (Figure 8), and the infection efficiency rate was assessed via flow cytometry. The results indicated that the efficiency rate of infection with an MOI of 200 was (53 ± 15)%, which was higher than (36 ± 9)%, the efficiency rate of infection with an MOI of 100.

### 3.7. hHGF Protein Expression in ADSCs

**Figure 9:**
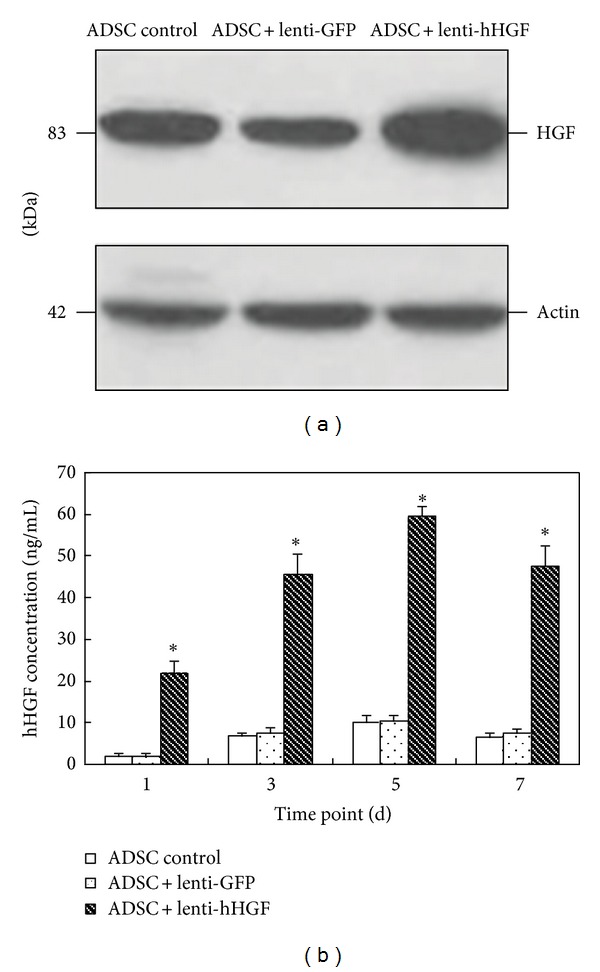
hHGF protein expressed in ADSCs. (a) Expression of hHGF as assessed by Western blot; (b) Enzyme linked immunosorbent assay of hHGF in the cell supernatants at different time points after infection. The results were expressed as mean ± standard deviation. *P < 0.001 for ADSC + lenti-hHGF versus ADSC control or ADSC + lenti-GFP.

The protein lanes corresponding to the uninfected ADSCs and the ADSCs infected with either lenti-hHGF or lenti-GFP are shown in Figure 9(a). The actin protein ran at 42 kDa, and the HGF protein ran at 83 kDa. Both the HGF gene-infected ADSCs and the uninfected ADSCs expressed HGF, but the expression level observed in the lenti-hHGF-infected group represented the highest level of expression of the three groups. ELISA found that both gene infected and uninfected ADSCs produced hHGF protein, but the concentrations were significantly higher in hHGF infected group at different time points after infection (*P* < 0.001). And the infected cells secreted the peak level of hHGF at 5 days after infection (Figure 9(b)).

## 4. Discussion 

Stem cells possess the ability to self-renew and differentiate into multiple cell types. Due to their reproducibility and multipotency, they have a significant role in many clinical and preclinical fields [11]. Stem cells can be harvested from various mesenchymal sources, such as bone marrow, peripheral blood, cord blood and adipose tissue. The most common source of stem cells is bone marrow. However, harvesting stem cells from bone marrow causes pain and discomfort to patients, and only a relatively small number of cells can be harvested.Currently, an increasing number of researchers are focusing on ADSCs because they are multipotent, immune-privileged, abundantly harvested, and easily expanded *ex vivo*. In the present study, ADSCs were isolated from adult human adipose tissue and subsequently amplified in culture. The cultured ADSCs were positive for CD29, CD44, and CD71 expression, but they did not express hematopoietic and endothelial lineage markers (CD31, CD34, CD45, CD106, and CD117). The phenotype of these cells was similar to that of the MSCs derived from bone marrow with the exception that MSCs express CD106. Additionally, the expression of immunogenicity markers (CD14, CD45, CD86, and HLA-DR) was not observed, which demonstrated that the human ADSCs were immune privileged. The osteogenic- and adipogenic-induced differentiation experiments confirmed that the obtained ADSCs possessed multiple differentiation abilities. 

Single-stem cell therapy is characterized by some deficiencies, such as the instability of stem cells, low cell numbers, and low activity. Therefore, stem cells combined with cytokines are more effective, and this combination has become the new therapy for IHD. HGF, which was originally identified and cloned as a potent for hepatocyte, has been reported to possess mitogenic, angiogenic, antiapoptotic, and antifibrotic activity in various cells. Many studies have shown that acute myocardial infarction, ischemia reperfusion injury, and congestive heart failureinduce the expression of HGF in the heart. HGFgene therapy can improve cardiac function via the induction of angiogenesis, reduction of fibrosis, and recruitment of stem cells derived from bone marrow, which affects myocardial regeneration [12]. To express HGF in the target cells, we selected a lentiviral vector for the gene therapy study because lentiviralvectorshave several attractive gene delivery vehicle properties as follows: (i) sustained gene delivery via stablevectorintegration into the host genome; (ii) the capability of infecting both dividing and nondividingcells; (iii) broad tissue tropisms, including important types of gene- andcell-targeted therapy; (iv) no viral protein expression aftervector transduction; (v) the ability to deliver complexgeneticelements, such as polycistronic and intron-containing sequences; (vi) a potentially safer integration site profile; (vii) a relatively easy system forvectormanipulation and production [13]. 

In this study, we constructed a HGF recombinant lentiviral expression vector. Using molecular cloning techniques, such as PCR and DNA sequencing, the human HGF lentiviral expression plasmid was successfully constructed, and high titer viral particles were obtained after plasmid transfection into packaging cells. The HGF-GFP expression levels reached more than 60% when human ADSCs were infected. The western blot and ELISA results showed that HGF gene-infected ADSCs overexpressed HGF. These results demonstrated that the lentiviral vector successfully delivered the human HGF gene into ADSCs and mediated high levels of HGF expression in ADSCs. In the future, ADSCs infected with the recombinant human HGF lentiviral vector will be used to study the function of the target gene in combination with stem cells in IHD and other ischemic diseases.
